# A Review on Biosensors and Nanosensors Application in Agroecosystems

**DOI:** 10.1186/s11671-021-03593-0

**Published:** 2021-08-30

**Authors:** Pankaj Sharma, Vimal Pandey, Mayur Mukut Murlidhar Sharma, Anupam Patra, Baljinder Singh, Sahil Mehta, Azamal Husen

**Affiliations:** 1 Department of Microbiology, CCS Haryana Agricultural University, Hisar, Haryana 125004 India; 2 National Institute of Plant Genome Research, Aruna Asaf Ali Marg, New Delhi, 110067 India; 3 Department of Agriculture and Life Industry, Kangwon National University, Chuncheon, Gangwon-do 24341 Republic of Korea; 4 International Centre for Genetic Engineering and Biotechnology, Aruna Asaf Ali Marg, New Delhi, 110067 India; 5 Wolaita Sodo University, P.O. Box: 138, Wolaita, Ethiopia

**Keywords:** Agroecosystems, Nanoparticles, Nanosensors, Biosensors, Pesticides, Heavy metals, Pathogens, Agricultural production

## Abstract

Previous decades have witnessed a lot of challenges that have provoked a dire need of ensuring global food security. The process of augmenting food production has made the agricultural ecosystems to face a lot of challenges like the persistence of residual particles of different pesticides, accretion of heavy metals, and contamination with toxic elemental particles which have negatively influenced the agricultural environment. The entry of such toxic elements into the human body via agricultural products engenders numerous health effects such as nerve and bone marrow disorders, metabolic disorders, infertility, disruption of biological functions at the cellular level, and respiratory and immunological diseases. The exigency for monitoring the agroecosystems can be appreciated by contemplating the reported 220,000 annual deaths due to toxic effects of residual pesticidal particles. The present practices employed for monitoring agroecosystems rely on techniques like gas chromatography, high-performance liquid chromatography, mass spectroscopy, etc*.* which have multiple constraints, being expensive, tedious with cumbersome protocol, demanding sophisticated appliances along with skilled personnel. The past couple of decades have witnessed a great expansion of the science of nanotechnology and this development has largely facilitated the development of modest, quick, and economically viable bio and nanosensors for detecting different entities contaminating the natural agroecosystems with an advantage of being innocuous to human health. The growth of nanotechnology has offered rapid development of bio and nanosensors for the detection of several composites which range from several metal ions, proteins, pesticides, to the detection of complete microorganisms. Therefore, the present review focuses on different bio and nanosensors employed for monitoring agricultural ecosystems and also trying to highlight the factor affecting their implementation from proof-of-concept to the commercialization stage.

## Introduction

The past several decades have witnessed a lot of challenges like perpetual demographic strain, unceasingly fluctuating climatic conditions, as well as the heightened sweepstakes for the resources, all of which have posed an egregious threat and thus provoked a dire need for guaranteeing global food security. The existing agricultural practices for fulfilling the food requirements include uncontrolled use of resources, sophisticated machinery as well as increasing and indiscriminate use of agrochemicals. These practices have led to significant deterioration of the soil, air, and water resources, thereby have expressively upturned the levels of pollution in the agricultural environments, which in turn has strongly affected human/animal health. The extent of health effects of pesticide use can be estimated from the information that 26 million people become victims of pesticide poisoning annually on a global basis which results in about 220,000 annual deaths [1]. Furthermore, due to their persistent nature, the residues of pesticides stay in the environment for a prolonged time period thereby contaminate the soil and thus raise concerns about the functioning of the soil, biodiversity, and food safety [2]. Moreover, there are many reports already available about the entry of pesticide residues in the food chain followed by their accumulation in the body of consumers which further results in severe health issues. The pesticides are also known to be cytotoxic and carcinogenic by nature [3–6]. They can also induce various nerve and bone marrow disorders, infertility, as well as respiratory and immunological diseases [7–10]. Therefore, the monitoring of pesticide residues in the environment becomes an imperative concern. Moreover, monitoring such residual pesticides regularly will also provide information about whether their occurrence is within or beyond the acceptable limits [11].

Another important challenge that is faced by the agroecosystems is the persistence of lethal heavy metals comprising cadmium, mercury, copper, zinc, nickel, lead, and chromium as they are held responsible for prolonged and significant damage to various biotic systems by disrupting biological actions at the cellular level [12, 13], for instance, via disruption of photosynthesis, disruption of mineral absorption, interruption of electron transport chain, induction of lipid peroxidation, disturbance in the metabolism of essential elements, induction of oxidative stress and by damaging the plant organs like root, leaves, and other cellular components [14–16]. Definitely, their natural occurrence in the earth’s crust is an undeniable fact but the uncontrolled anthropogenic activities have disturbed the geochemical cycling and biochemical balance of these elements to a remarkable extent. This has resulted in an increased prevalence of such metals in different plant parts. Together, all the risks posed by the presence and prevalence of heavy metals in various ecosystems emphasize the need to develop systems for sensing them even at low concentrations in environmental samples [17].

**Fig. 1:**
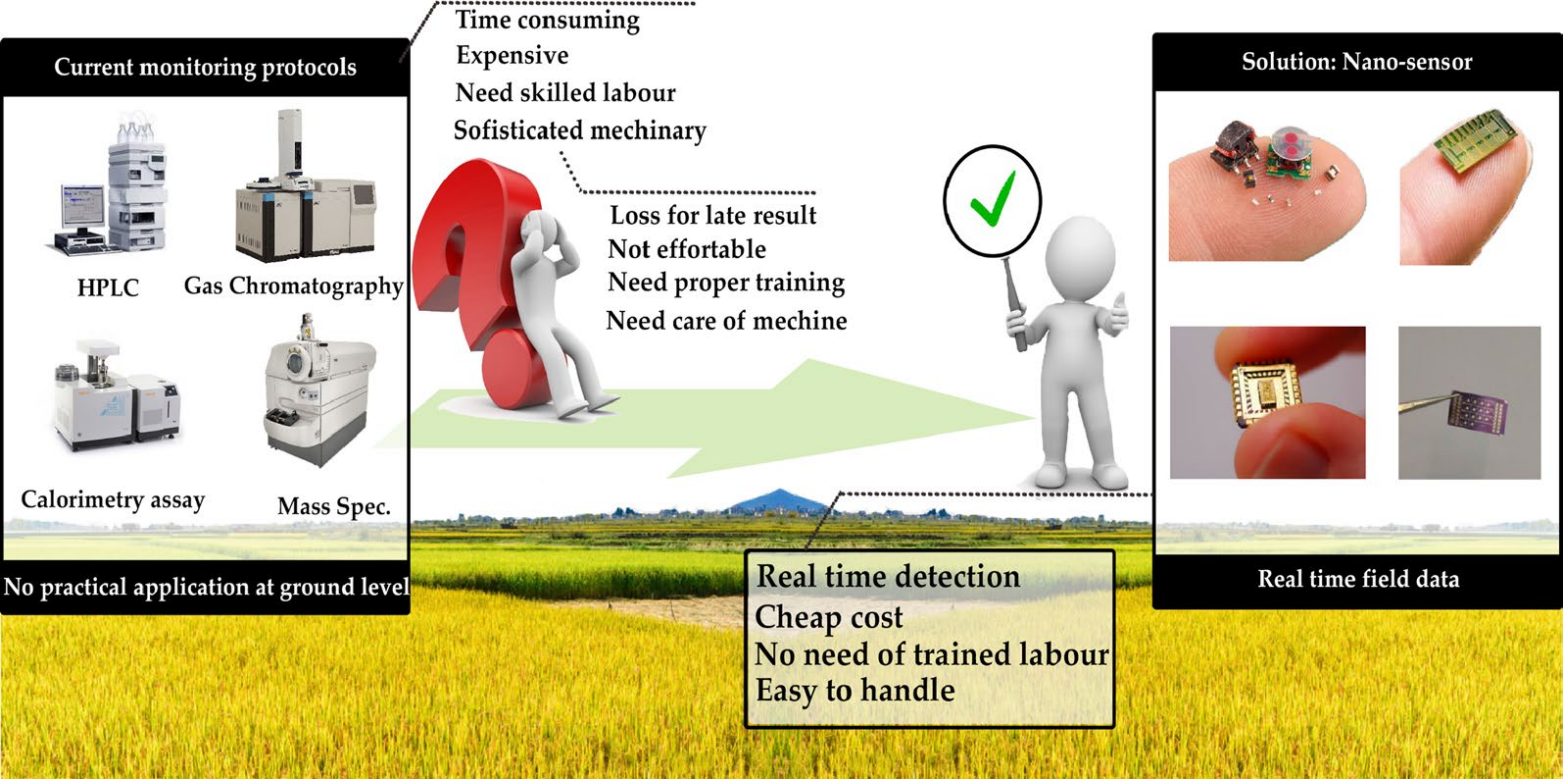
Schematic representation highlighting the differences between traditional and advanced monitoring technologies

**Fig. 2:**
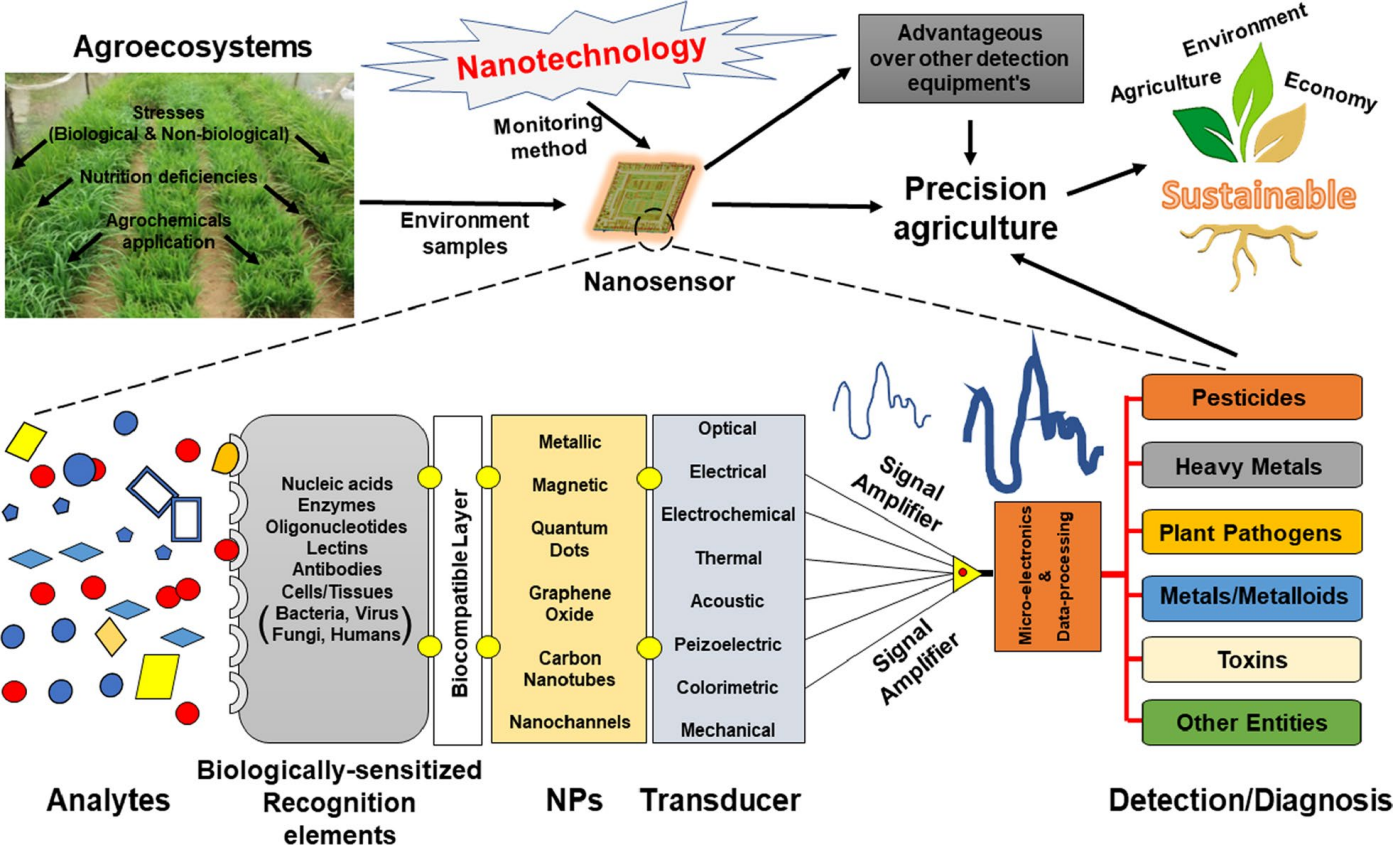
Simplified representation illustrating the component of nanosensors to monitoring agroecosystems

At present, various methods available for monitoring agroecosystems include gas chromatography, high-performance liquid chromatography, mass spectroscopy, and more (Fig. 1). All these techniques can easily detect and quantify contaminants in the environment as well as agricultural samples. On the contrary, the sensitivity, specificity, and reproducibility of such measurements are incontrovertible but the deployment of these methods is predominantly restricted by their time consumption, high cost, and requirement of sophisticated appliances along with skilled personnel [8]. Therefore, there is an impenetrable need for modest, quick, and economically viable methods for monitoring such agricultural contaminants [18–20]. Nanosensors are nanoscale element devices that are engineered to identify a particular molecule, biological component, or environmental circumstances. These sensors are highly specific, handy, cost-effective, and detect at a level much lower as compared to their macroscale analogs. A typical nanosensor device operation contains three basic components:

1. Sample preparation: It could be a homogenous or complex suspension of gas, liquid or solid-state. Sample preparation of agroecosystem is very challenging due to impurities and interferences. The sample contains specific molecules, functional groups of molecules or organisms, that the sensors can target. These targeted molecules/organisms known as the analyte and could be molecules (dyes/colors, toxicants, pesticides, hormones, antibiotics, vitamins, etc.), biomolecules (enzymes, DNA/RNA, allergens, etc.), ions (metals, halogens, surfactants, etc.), gas/vapor (oxygen, carbon dioxide, volatile compounds, water vapors, etc.), organisms (bacteria, fungi, viruses) and environment (humidity, temperature, light, pH, weather, etc.)
2. Recognition: Certain molecules/elements recognize the analytes within the sample. These recognition molecules are antibody, aptamer, chemical legends enzymes, etc., and having high affinity, specificity, selective characteristics to their analytes to quantify them to acceptance levels.
3. Signal transduction: Certain signal transduction methods have categorized these modest devices into different types such as optical, electrochemical, piezoelectric, pyroelectric, electronic, and gravimetric biosensors. They convert recognition events into computable signals that are further processed to produce the data (Fig. 2).

The nano-technological interventions position the stimulus to transfigure the diverse zones of diagnostics like health, medication, food, environment, as well as the agriculture sector, thereby, transitioning the speculative characteristics into the practical output [21–28]. Nanotechnology plays a significant role in the advancement of numerous diagnostic methodologies by rendering mankind with contemporary tools comprising of sensors established on bio-techniques, nano-based medical facilities, along with bio-photonics which simplifies the detection of pesticides, drug residues, food-borne pathogenic microorganisms, toxin contaminants, and heavy metal ions [24, 29]. Fortunately, the arena of nanotechnology comprises an understanding coupled with governing material at the atomic or molecular scale where matter unveils distinctive attributes and performances when equated to the bulk form of similar matter [30]. Currently, among all the approaches, a biosensor is a modest and compacted investigative device that has the capability of producing definite systematic data either in a quantitative way or in a semi-quantitative form by employing a recognition component of biological origin which is joined to a signal transformation unit [31–33]. The type of employment of the signal transduction method has categorized these modest devices into different types such as optical, electrochemical, piezoelectric, pyroelectric, electronic, and gravimetric biosensors [34]. The recent advances in nanotechnology have opened various new ways for designing biosensors [29, 35]. The hybridization of nano-materials with different biosensing daises (nano-bio sensors) offers a great deal of conjoining and multipurpose approaches for enhanced sensitivity for detection [36] and thereby improves the capability in the monitoring of even a single molecule [32, 37, 38]. The nanoscale has been defined approximately as 1–100 nm, which is also equivalent to a billionth part of a meter. It can be easily understood by comparing it with the dimensions of an average bacterial cell which is around 1000 nm in diameter [39]. The nanomaterial that is employed in sensing is called a nanosensor which is constructed at the atomic scale for data collection. The nanomaterial is further reassigned into information which can be analyzed for several applications, for instance, to keep an eye on various physical and chemical portents in areas hard to approach, detect different chemicals of biological origin in various cellular organelles, and determine particles of nanoscale in the environment and the industry [40, 41]. The presence of even a single virus particle and substances present in very low concentrations can be detected using nanosensors. A nanosensor is comprised of a bio-sensitive layer that is attached covalently to another element called a transducer. The physiochemical change produced due to the interactions of the target analyte with the bioreceptor is converted into an electrical signal [40].

**Fig. 3:**
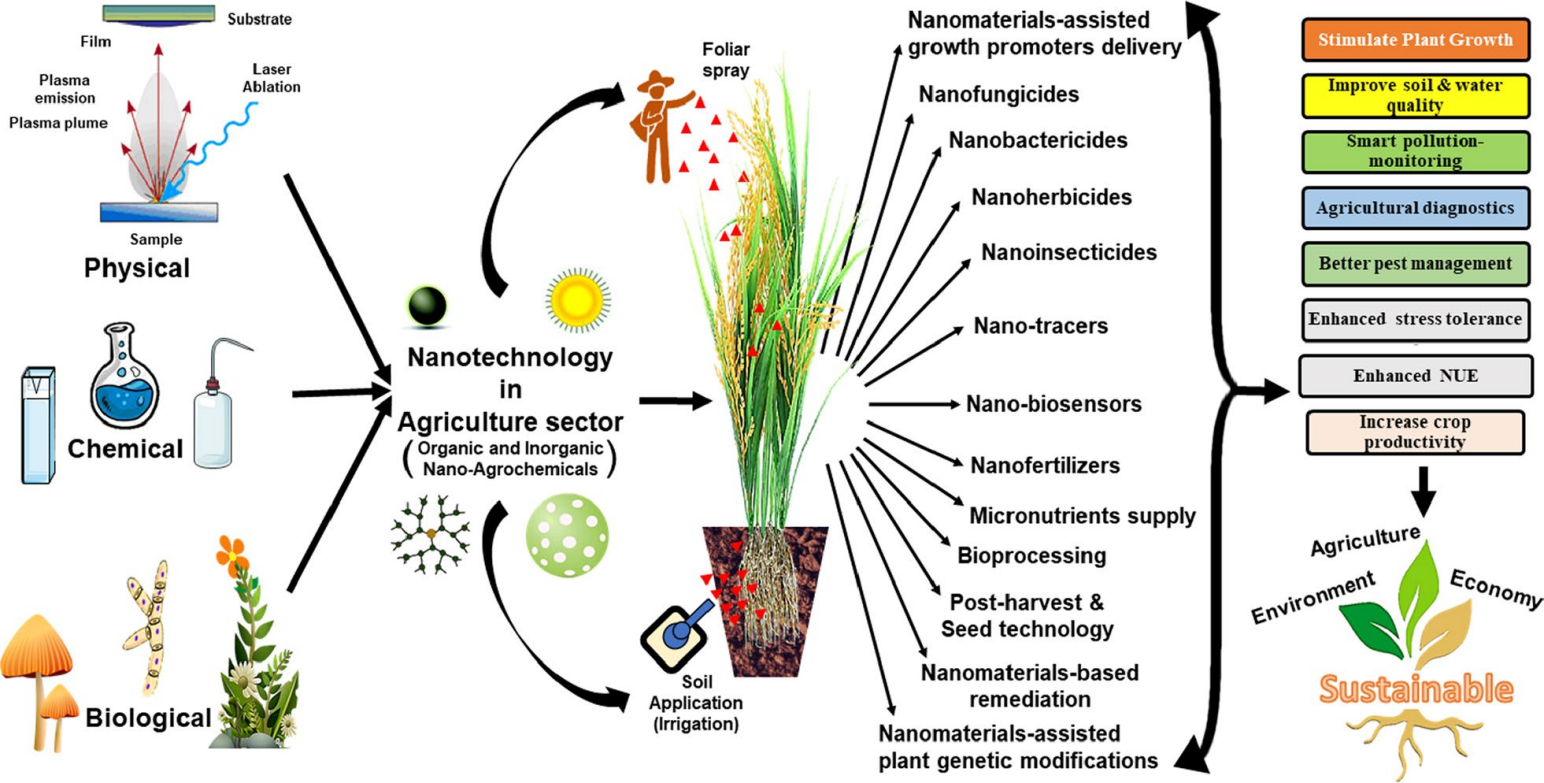
Various applications of nanotechnology in the agriculture sector

In recent years, a great deal of superior visual recognition bio and nanosensors have been employed for the detection of several composites from a vast array of samples. The range of composites covers several metal ions, proteins, pesticides, antibiotics to the detection of complete microorganisms, and nucleic acid amplification and sequencing [19, 33, 42, 43]. Apart from monitoring the agricultural-controlling process and residues, other potential applications of nanotechnology have also been surfaced in the last two decades [44–47]. The imperative benefits for engaging nanotechnology in the improvement of the agriculture sector include nanomaterials-assisted delivery of growth promoters [44, 48, 49], nutrition (especially micronutrients) [49, 50] as well as genetic modifications in plants [51, 52]. Additionally, various pesticides in form of nanofungicides, nanobacteriocides as well as nanoinsecticides have been also found to be employed [50, 53–55]. Furthermore, other benefits of nanotechnology include nanomaterials-based remediation [56], nanoherbicides [57] as well as uses in bioprocessing [58], aquaculture [59], post-harvest technology [60], veterinary care [61], fisheries [62], and seed-technology [63]. All these applications together show various advantages like reduced pollution (mainly soil and water), reduction in related costs of environmental protection, and enhanced nutrient use efficiency [45, 46, 50, 56, 64–68] (Fig. 3). Given the above-mentioned facts, the present review targets the employment of different kinds of nanosensors in different agroecosystems for revealing different components along with the detection of some foreign components intruding the natural agroecosystems.

## Nanosensors for Pesticide Detection

**Fig. 4:**
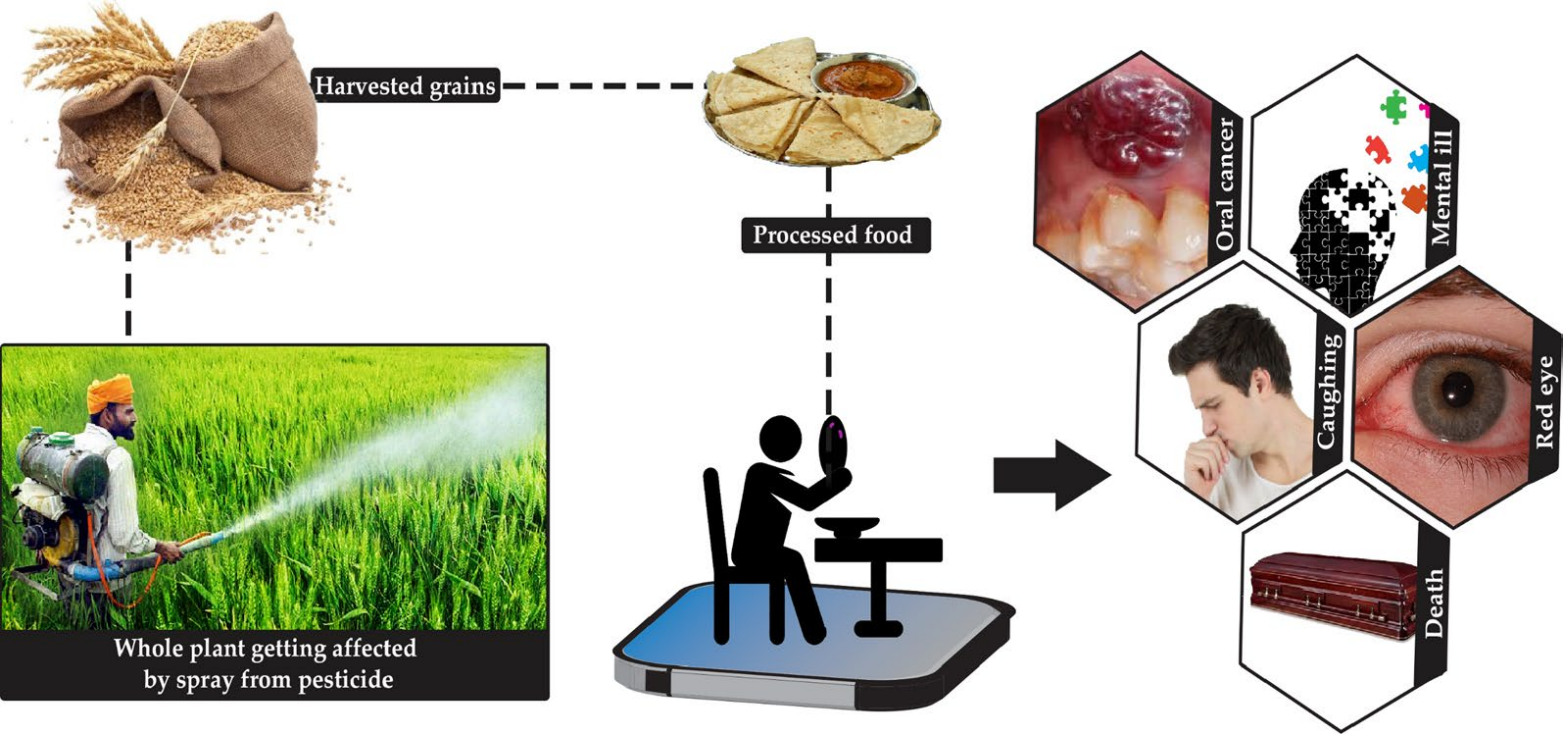
Adverse effects of pesticides on human health

Pesticides find broad applications in agricultural systems for the avoidance, regulation, or abolition of pests, insects, weeds, and fungi to increase the productivity of agroecosystems [69]. The use of pesticides is on a perpetual increase and they might secure almost one-third share of the global agricultural products [70]. However, the indiscriminate usage of pesticides at field conditions has contaminated the groundwater and marked their accumulation in the food resources, thereby has also seriously affected non-target species like human beings and animals (Fig. 4). The exposure of humans to pesticides can affect health in diverse ways and the attendant health effects produced can range from mutagenicity, neurotoxicity, carcinogenicity to genotoxicity [71, 72]. Some pesticides like organophosphates accrue in the animal bodies even with their application in a small concentration and exposure to higher concentrations leads to the inhibition of enzymes like acetylcholinesterase that impart severe health risks to humans [73]. Therefore, to ensure food safety, the development of superior methods of detecting pesticide residues is very important.

Although various approaches are being used from a very long time for the detection of pesticide residues like high-performance liquid chromatography, colorimetric assays, enzyme-linked immune sorbent assay, liquid/gas chromatography-mass spectrometry, electrophoresis, and fluorimetric assay procedures [8, 74–79]. Nevertheless, the majority of these techniques are single-signal assays that require costly apparatus, professional operators, and complex pretreatment of the samples whereas some are even prone to variations in the environmental conditions [80, 81]. Therefore, such detection measures are not suitable for the on-site detection of residual pesticides. Additionally, they are also not found to be appropriate for real-time detection which constraints their use in emergency cases [82]. Consequently, detection methods employing multiple signals enhance the reliability and convenience of the analysis. For instance, methods targeting a combination of a multi-signal fluorimetric method with colorimetric assays are capable of circumventing the influence of background in multifaceted structures and complement naked-eye sensing in different practical solicitations [83]. Therefore, concentrating more effort in evaluating different approaches for the detection of pesticides in a speedy, simplistic, selective, delicate, precise, and comprehensible means has led to the development of optical sensors for detecting pesticide residues [80].

Numerous optical strategies have already been recognized for pesticide detection which exploited recognition elements like enzymes, antibodies, molecularly imprinted polymers, aptamers, and host–guest recognizers. Such approaches can staunchly recognize and detect the particular pesticidal particle [81, 84–88]. Furthermore, the coupling of recognition components with the nanomaterials results in greater levels of sensitivity and tremendous specificity for instantaneous deployment, which is a principal requirement for expeditious and efficacious pesticide detection [82]. So the quest for a prompt, sensitive, specific, precise, and easy to operate method for detecting residual pesticides has resulted in the deployment of nanosensors as a pre-eminent substitute to conventional methods due to their cost effectiveness, compactness, ease of transportation, extraordinary sensitivity, and a lesser time of detection [89] (Fig. 1).

In general, an optical sensor is composed of a recognition element that is specific for the particular residual pesticidal particle and can network with the other constituent, the transducer, which is employed to produce the signal for the binding of a particular pesticide residue to the sensor. The recognition components which are comprised of enzymes, antibodies, molecularly-imprinted polymers, aptamers, and host–guest recognizers, are gripping the consideration of the scientific community for improving the diagnostic performance of any sensor. The prevailing entrenched optical probes could be categorized into four types based on signal output formats. These are fluorescence (FL), colorimetric (CL), surface-enhanced Raman scattering (SERS), and surface plasmon resonance (SPR) optical sensors [90].

Another kind of nanosensors widely known are immunochromatographic strip (ICTS) nanosensors that are broadly accredited in point-of-care analytical devices [91]. The immunochromatographic assays have also been reported for their involvement in monitoring agroecosystems owing to their point-of-care testing behavior. For instance, a visible colorimetric readout strategy was adopted in the reported immunochromatographic assay for the detection of GM crops, which only provided a yes/no response and often suffered from insufficient sensitivity [92–94]. Similarly, the gold nanoparticle-based ICTS sensors have also been reported to possess low detection sensitivity, owing to the production of relatively weaker color density, which limits their application [95, 96]. However, their sensitivity can be improved by several proposed amplification strategies like augmenting detection signal intensity, enhancing the affinity of the reagent, optimizing the labeling techniques, and amending the shapes of strip devices [96]. Therefore, the improved ICTS nanosensors can also prove to be an economically viable tool for pesticide residue detection in agroecosystems.

The amalgamation of nanotechnology with different electrochemical approaches compromises a superior operational surface area to the sensor along with a decent check on the electrode micro-environment. Nanoparticles owe divergent and numerous properties thereby possess the potential to play multiple purposes in the sensing structures grounded on electrochemical phenomena, for instance, catalyzing the electrochemical reactions, enhancing the transfer of electrons, tagging, and performing as a reactant [97]. Therefore, electrochemical nanosensors appear to be an effective tool meant for pesticide detection. Recently, electrochemical biosensors that were primarily grounded on the enzyme cholinesterase appeared as propitious devices meant for detecting residual pesticidal particles especially belonging to the class carbamates and organophosphates attributable to their great perceptiveness, choosiness, and painless methods of creation [98, 99]. Nevertheless, enzyme-based biosensors undergo quite a lot of restrictions comprising high price, diminished activity of the enzyme, and truncated reproducibility [100]. Moreover, enzymes seem to be inherently unstable and are also subject to denaturation in hostile environmental conditions which restricts the lifetime of biosensors thereby limiting their practical applications [101]. Additionally, a manifestation of several impurities such as the occurrence of different heavy metals in the samples of biological origin can also disturb the selectivity as well as the sensitivity of the enzyme during the detection that may produce false-positive results [102]. Therefore, it provokes the need for non-enzymatic electrochemical biosensors. Nanomaterials appear to be promising contestants to formulate non-enzymatic electrochemical sensors [103]. Various categories of nanomaterials comprising nanoparticles (e.g., CuO, CuO–TiO_2_, and ZrO_2_, NiO), nanocomposites (such as molybdenum nanocomposite), and nanotubes (e.g., peptide and carbon nanotubes) are widely found to be engaged in electrochemically determining the residual pesticidal particles [104–106]. The explicit and profound investigation of the residual pesticidal particles by such nanomaterials is attributable to their extremely small size, greater surface area, and the possession of inimitable electrical as well as chemical properties [70].

The sensitivity, as well as selectivity of various nanosensors for definite pesticides, has been reported in various studies (Table 1), for instance, the two different optical sensors grounded on silver nanodendrites and upconverting nanoparticles were found to detect the pesticides dimethoate and metribuzin at the levels of 0.002 ppm and 6.8 × 10^−8^ M, respectively [107, 108]. Similarly, the electrochemical nanosensor grounded using CuO nanoparticles decorated with 3D graphene nanocomposite detected malathion at the level of 0.01 nM [109] whereas the electrochemical aptasensor fabricated through chitosan-iron oxide nanocomposite detected malathion at a surprising sensitivity of 0.001 ng/mL [110].

**Table 1:** Highlights of nanosensors for pesticide detection

Nanosensor type	Used nanomaterial	Pesticide detected	Limit of detection	Method of nanosensor Formulation	Sensing mechanism	Observation	References
Fluorescent-nanosensor	3-aminopropyl-triethoxysilane coated Yb_2_O_3_	Imazapyr	0.2 ppm	Hydrothermal method	Quenching of fluorescence intensity for APTES coated Yb_2_O_3_ NPs with the increasing concentration of imazapyr	Among the lanthanide oxide based nanomaterials, ytterbium (III) oxide (Yb_2_O_3_) NPs owes unique optical and luminescence properties with excellent efficiency for real field conditions	[173]
Surface plasmon resonance (SPR) based affinity sensor	Atrazine imprinted nanoparticles	Atrazine	0.7134 ng/mL	Atrazine imprinted nanoparticles synthesis using emulsion polymerization method followed by their attachment on the gold surface of SPR	Increase in resonance frequency in proportion to the increment in atrazine concentration	The plastic antibody-based SPR nanosensor is an attractive recognition element for the detection of atrazine with high selectivity and sensitivity	[174]
Surface plasmon resonance based fiber–optic nanosensor	Tantalum(V) oxide nanoparticles	Fenitrothion	38 nM	Chemical synthesis of Ta_2_O_5_ nanoparticles embedded in reduced graphene oxide matrix followed by its adhesion on silver-coated fiber optic probe	Change in refractive index due to the interaction of fenitrothion with the silver film	The sensor is selective, repeatable and works at ambient temperature with a response time of 23 s	[175]
Fluorescence sensor	Copper (II) oxide and multiwall carbon nanotubes (MWCNTs)	Glyphosate	0.67 ppb	CuO/MWCNT were prepared by precipitating copper nitrate by the addition of aqueous NaOH solution	Inhibition of the catalytic activity of CuO/MWCNTs	A highly selective & promising approach for rapid screening of glyphosate	[176]
Electrochemical Luminescence sensor	Luminol-gold nanoparticles-L-cysteine-Cu(II) composites	Glyphosate	0.5 nM	Layer-by-layer assembly of graphene-gold nanoparticle composite and Lu-Au-Lcys-Cu(II) composite	Decrease in electrochemical luminescence intensity with a respective increase in the glyphosate concentration	The sensor worked on dual inhibition strategy with excellent detection performance, high sensitivity, desirable reproducibility, stability, and accuracy	[177]
Electrochemical sensor	CuO-TiO2 hybrid nanocomposites	Methyl parathion	1.21 ppb	CuO-TiO_2_ nanocomposites prepared by a facile liquid-control-precipitation method were decorated on the glass carbon electrode	Differential pulse voltammetry measurements assessed from decline in current density with increase in the methyl parathion concentration	A non-enzymatic sensor with good stability and excellent reproducibility	[103]
Electrochemical aptasensor	Chitosan-iron oxide nanocomposite	Malathion	0.001 ng/mL	Iron Oxide nanoparticles synthesized using chemical co-precipitation method were deposited on fluorine tin Oxide followed by the immobilization of aptamer onto the iron oxide doped-chitosan/FTO electrode using streptavidin	Decline in the Differential Pulse Voltammetry peak current of the aptaelectrode with a corresponding increase in malathion concentration due to the formation of more 3D-complex between aptamer with malathion	A very attractive alternative to quantify and monitor malathion due to its sensitivity, stability, short analysis time and cost-effectiveness	[110]
Electrochemical nanosensor	CuO nanoparticles decorated 3D graphene nanocomposite	Malathion	0.01 nM	Copper oxide nanoparticles electro-catalyst was prepared on 3D graphene synthesized using hydrothermal process	Decline in peak current with the increasing concentrations of malathion	Highly sensitive, reproducible and applicable in real field conditions	[109]
Optical nanosensor	Silver nanodendrites	Dimethoate	0.002 ppm	Ag nanodendrites fabricated by laser-assisted photochemical method were immobilized on the surface of microsphere end-shape optical fibre	Increase in the intensity of the surface-enhanced Raman spectroscopy (SERS) signal with a proportionate increase in dimethoate concentration	A direct, rapid, real-time and non-destructive method of detecting pesticide residue in the outdoor fields	[107]
Optical sensor	Upconverting nanoparticles (UCNPs) of the NaYF_4_:Yb, Er type	Metribuzin	6.8 × 10^−8^ M	Upconverting nanoparticles synthesized using the coprecipitation method of lanthanide metal-EDTA complexes were later used in the preparation of the sensor film by dissolving UCNPs in tetrahydrofuran along with the incorporation of NIR dye, PVC polymer, dioctyl phthalate	Metribuzin changes the color of sensor film from green to blue with a significant blue shift in the absorption peak	Highly sensitive sensor with unique luminescence properties of UCNPs and great recognition abilities within a very low detection limit	[108]

## Nanosensors for Detection of Heavy Metals

The existence of diverse heavy metal ions like Pb^2+^, Hg^2+^, Ag^+^ , Cd^2+^, and Cu^2+^ from different resources has a precarious influence on human beings as well as their surroundings. The accretion of heavy metals in different environments is supported by the uninterrupted boost in the agricultural and industrial accomplishments along with the inadequate discharge of heavy metal ions from wastewaters and domestic emissions [111]. Therefore, to assure the security of the environment along with the health analysis, the ferreting out of the trace heavy metal ions through proficient practices is extremely desired. The apprehension of heavy metals can be accomplished by exploring several analytical systems [112], for instance, X-ray fluorescence spectrometry (XRF), atomic absorption spectrometry (AAS), atomic emission spectrometry (AES), and inductively coupled plasma mass spectrometry (ICP-MS) but their application suffers a lot of limitations like lavishness of devices, time-consuming methods, and labor intensiveness. Therefore, to guide these restrictions, numerous types of optical, electrochemical, and colorimetric stratagems have been comprehensively scrutinized (Table 2) to contrive modest and lucrative daises for apprehending delicate, hasty, and discerning exploration of heavy metal ions [113, 114].

**Table 2:** Recent developments in nanosensors for the detection of heavy metals

Nanosensor type	Used nanomaterial	Detected heavy metal	Limit of detection	Method of nanosensor formulation	Sensing mechanism	Observation	References
ICTS Nanosensor	Au	Cadmium	0.35 µg/L	The Cd(II)-EDTA-BSA antigen and goat anti-mouse IgG were dispersed on the Nitrocellulose (NC) membrane followed by the addition of concentrated colloidal gold probe on glass fiber membrane	Decline in color intensity with the increase in the concentration of Cd(II)	A highly sensitive sensor specific for the detection of cadmium	[178]
ICTS Nanosensor	Au	Lead	0.19 ng/mL	Au nanoparticle conjugates were prepared using anti-Pb(II)-ITCBE monoclonal antibody and colloidal gold solution. The lateral flow assay strip for detecting lead ions was constructed using an NC membrane, absorbent pad, and two conjugate pads	Decline in color intensity with the increase in the concentration of Pb(II)	The detection method could be accomplished within 15 min	[179]
Optical	Nanohybrid CdSe QDs	Cadmium	25 nM	Amino capped CdTe@SiO_2_ core–shell structured fluorescent silica NPs synthesized using a modified reverse microemulsion method. The green-emitting dual-stabilizers capped CdSe QDs were covalently linked to the silica surface to form CdTe@SiO_2_@CdSe ratiometric probes	On the cadmium introduction the green photoluminescence got gradually restored	An alternative sensing approach for highly sensitive and selective detection	[117]
Colorimetric nanosensor	Mesoporous silica nanoparticles (MSN)	Mercury	60 pM	The nanodevice was fabricated by implanting new dithiocetal-grounded stimulus receptive molecular gates on MSN loaded with a reporter dye	Hg(II) has high affinity for sulfur, and can disrupt the linear dithioacetal linkages upon interaction with solid S_2_, yielding new Hg(S-R)_2_ leading to subsequent cargo release	The nanosensor displays a sensitivity of 29.9 a.u/μM	[180]
Colorimetric nanosensor	Au	Pd(II)	4.23 µM	Gold nanoparticles were stabilized by cationic 1-(3-(acetylthio)propyl)pyrazin-1-iumligand to detect Pd(II)	Pd(II) selectively induced the aggregation of APP-AuNPs as compared to other metals, resulting into the complete significant disappearance of surface plasmon resonane	The nanosensors warrants naked eye detection	[181]
Colorimetric nanosensor	Silver-coated gold nanobipyramids	Mercury	0.8 µM	The Au NBs were synthesized as per the seed-mediated growth method and later Au NBs@Ag nanoparticles were synthesized by adding different volumes of AgNO_3_ to Au NBs colloidal solution	Hg^2+^ detection is achieved by etching silver-coated gold nanobipyramids which brings a color change	The method is devoid of tedious procedures and is time-saving	[182]
Multimodal nanosensor	Superparamagnetic Fe_2_O_3_ nanoparticles	Mercury	0.49 nM	Fe_2_O_3_ nanoparticles prepared by the chemical coprecipitation method were coated by silica and later electrostatically attached with the cysteamine capped CdTe QDs	Fluorescence quenching with increasing concentrations of Hg^2+^	The detected analyte can be removed with the use of an external bar magnet leaving no residual pollution	[124]
Surface plasmon resonance	Epicatechin coated silver nanoparticles (ECAgNPs)	Lead	1.52 μM	The ECAgNPs, were prepared by mixing various ratios of AgNO_3_ and epicatechin followed by magnetic stirring and was later used for lead detection	The metal exhibited hyperchromic shift upon binding with epicatechin based silver nanoparticles	ECAgNPs can selectively detect Pb^2+^ even in the presence of other interfering metal ions	[126]
Electrochemical sensor	Nano sheets of Fc-NH_2_-UiO-66, and thermally reduced graphene oxide (trGNO)	Cadmium, Lead and Copper	8.5 nM for Cd^2+^, 0.6 nM for Pb^2+^ and 0.8 nM for Cu^2+^, respectively	NH_2_-UiO-66 was synthesized by hydrothermal method whereas N-hydroxysuccinimide (NHS) and 1-ethyl-3-(3-dimethylaminopropyl) carbodiimide (EDC) were used as crosslinking agents to prepare Fc-NH2-UiO-66 followed by the dispersion of Fc-NH 2—UiO-66 on the trGNO nanosheets	The peak current increases with the increasing concentrations of heavy metal	A very good platform for simultaneous detection of multiple heavy metal ions	[183]
Magnetic-fluorescent based nanosensor	Carboxymethyl chitosan-functionalized magnetic-fluorescent nanocomposites	Mercury	9.1 × 10^−8^ mol/L	Carboxymethyl chitosan was used as encapsulation agent to package Fe_3_O_4_ nanoparticles and QDs, resulting in the multifunctional magnetic-fluorescent nanoparticle which were later used as nanosensors	Quenching of nanosensor’s fluorescence	The nanosensor shows a superior selectivity and sensitivity for Hg^2+^ ions	[128]

Optical chemical sensors that are frequently targeted for heavy metal detection fit into a cluster of chemical sensors that primarily employ electromagnetic radiation for engendering a diagnostic signal in an element known as the transduction element. The interactions between the sample and the radiation change a specific optical consideration that can be interrelated to the concentration of an analyte [115, 116]. For instance, the optical nanosensor synthesized using nanohybrid CdSe quantum dots for the detection of cadmium restored its green photoluminescence on the sensation of cadmium metal [117]. The optical chemical sensors work on the principle of seemed variations in the optical possessions (emission, absorption, transmission, lifetime, etc.) which appear as a result of binding of the arrested indicator (organic dye) with the analyte [118]. The approach of enticing graphene-based nanotechnology embarks as an attributable tool that incapacitates such challenges and bequeaths the sensing platform with enhanced performance. The optical techniques predominantly grounded on nanomaterials of graphene-origin have been advanced in recent times as one of the rousing practices for detecting heavy metal ions owing to the probable eminences of their meek construction and sentient appreciation of some distinctive metal ions [116].

The noble nanoparticles like Ag, Au, Pd are endowed with a unique trait of mimicking peroxidase activity, and their congregation with graphene boosts their sturdiness along with superior catalytic performance. There is a diverse magnitude of sensors concerned with the detection of numerous heavy metal ions based on this feature. The hybridization of graphene oxide with silver nanoparticles resulted in nanohybrids mimicking the peroxidase enzyme activity and they were further found to be able to discriminate amid double-stranded and single-stranded DNA molecules. Therefore, making the calorimetric detection of Pb^2+^ and Hg^2+^ suitable based on the metal ion-provoked change in the DNA conformation because the conformation was altered into either a quadruplex arrangement or a hairpin-like assembly in their occurrence [119, 120]. Moreover, such colorimetric approaches are advantageous due to their simple operation, economically feasible, transportable instrumentation, and easy-to-use applications. The chemosensors for detecting heavy metals are found to be troublesome for the elimination of the objective species as they would result in secondary pollution. Therefore, the integration of fluorescent and magnetic functionality together in a sole nanocomposite particle seems to be a capable substitute [121]. Nevertheless, the manifestation of the magnetic nanoparticles strongly quenches the photoluminescence of the fluorescent moiety, thus ascend a staid challenge towards the development of such kinds of nanocomposites. Therefore, to steer this concern, numerous interactions happening at the molecular level, such as hydrophobic and electrostatic interactions, hydrogen bonding, and covalent bonding are often targeted for nanocomposite synthesis. For instance, the quantum dots placed on the shallow of polymer-layered Fe_2_O_3_ globules by employing the approaches of thiol chemistry. The gold nanoparticles arrested on the surface of several materials including Fe_2_O_3_ nanoparticles, and the silica microspheres employing electrostatic connections have also been synthesized [122, 123].

The approach of synthesizing multimodal nanosensors using principles of nano-chemistry is rather more appealing as it not only efficiently detects but also removes the heavy metal ions in the aqueous media. The multimodal nanosensor synthesized by Satapathi et al. [124] through multistep production practice, entailed a thin silica shell that encapsulated the magnetic (Fe_2_O_3_) nanoparticles, an immovable spacer arm, and a fluorescent quantum dot meant for the coinciding recognition as well as the elimination of the spotted mercury ion. The exceptional sensitivity of this nanosensor can be marked by its capability of detecting Hg^2+^ at the nanomolar level with a limit of detection of just 1 nm. The eco-friendly aspect of nanosensor can be advocated by the unique attribute of removing the detected analyte by using an external bar magnet thereby leaving no leftover as a pollutant. Several compounds are used for stabilizing nanosensors, such as polysaccharides citrates, different polymers, and proteins to improve the attributes of the nanosensors [125]. The silver nanoparticles stabilized with epicatechin can be used for discerning detection of Pb^2+^, that too, in the occurrence of different snooping metal ions. The low limit of detection, easy synthesis, admirable discernment, and economical production, make ECAgNPs, a potent sensor destined for repetitive checking of Pb^2+^ intensities in the ecological models [126]. The employment of quantum dots offers remarkable advantages in terms of their photophysical as well as chemical attributes, thereby, making fluorescent quantum dots-based sensors an efficient tool for sensing numerous metal ions [127, 128]. However, the major disadvantage with the employment of quantum dots is their separation and recovery in practical applications which happens to be an immoderate, laborious, and tedious task. Nevertheless, the introduction of magnetic nanomaterials (Fe_3_O_4_) into the quantum dot-based fluorescence sensors solves this problem and offers several additional advantages owing to their high specific surface area, special magnetic properties, magnetic operability, and low toxicity. Yang et al. [128] established multifunctional magnetic-fluorescent nanoparticles grounded on the carboxymethyl chitosan amalgamated with fluorescent quantum dots and magnetic nanomaterials which could detect and separate Hg^2+^ simultaneously along with a sensing level of 9.1 × 10^−8^ mol/L. Thus, the unpretentious and sophisticated methodology of nanotechnology offers a direction concerning field-based heavy metal sensory devices in the future which now appears to be a difficult task along with various limitations.

## Nanosensors for Detecting Plant Pathogens

The ascertainment, recognition, and assessment of pathogens are vital for scientific elucidation, ecological surveillance, and governing food security. It is imperative for investigative outfits that the delicate element of biological origin, which is a constituent of biological provenance or biomimetic constituent, interacts with the analyte in the examination. There are numerous profound, trustworthy, and swift recognition components, for instance, lectin, phage, aptamers, antibody, bacterial imprint, or cell receptor, which have been described for exposure of bacteria [129]. The most widely used biosensing components for analyzing pathogens are bacterial receptors, antibodies, and lectins. These constituents find wide applications as biosensing components to scrutinize pathogens owing to their adaptability of amalgamation into biosensors [130, 131]. Aptamers, the nucleic acids having only a single strand, are economically feasible and chemically steady, as compared to the recognition elements which are based on the antibodies for detecting bacteria [132]. However, they also pose various disadvantages like batch-to-batch variations, sturdiness in complex materials and they are also comparatively complex to prepare. The approach pointing to ‘chemical nose’ is a recently established equipment for detecting pathogens. It appoints multifarious discriminatory receptors that generate a unique response configuration for every objective, thus permitting their ordering. It functions in a fashion analogous to the working of our intellect of smelling something [133]. This technique involves the training of sensors with competent bacterial samples to establish a reference database. The identification of bacterial pathogens is done by equating them with the reference catalog [134]. Usually, nanoparticle-centered “chemical nose” biosensors necessitate the amendment of the surface of the nanoparticle with several ligands where an individual ligand is liable for a distinctive communication with the objective [133]. The variance in the size, as well as the external make-up of the nanoparticles, is selected in a way that every single set of particles can retort to different classes of bacteria in an inimitable way thereby offers supplementary features to the absorption spectrum.

The addition of nanoparticles to the bacteria leads to the development of aggregates encompassing the bacteria as a result of electrostatic interfaces amid the anionic sections of the bacterial cell walls and cationic cetyltrimethylammonium bromide (CTBr). This process of aggregation promotes a change of color induced by a swing in localized surface plasmon resonance. The color variation is further denoted by procuring an absorption spectrum in the existence of several bacteria [135, 136]. The components of the bacterial cell wall which are responsible for this kind of aggregation are teichoic acids in Gram-positive and lipopolysaccharides and phospholipids in Gram-negative bacteria [137]. These aggregation patterns are unique and are motivated by the occurrence of extracellular polymeric substances on the bacterial surface. These varying aggregation patterns are accountable for offering discernable colorimetric responses. Therefore the “chemical nose” established on nanoparticles could be accomplished to sense blends of varying bacterial species. During infections the “chemical nose” is potent enough to differentiate amid polymicrobial and monomicrobial cases, which facilitates superior effectiveness along with prompting antimicrobial therapy, precluding the requirement of extensive and prolonged testing of the sample [133]. The multichannel nanosensors are highly sensitive and can detect bacterial species even strains present in biofilms within minutes. Li et al. [138] established a multichannel sensor based on gold nanoparticles (AuNPs) and used it to spot and recognize biofilms based on their physicochemical attributes. The sensitivity of the nanosensor can be well advocated by its ability to discriminate amongst six biofilms. Another sensor which was designed based on hydrophobically employed gold nanoparticles by Phillips et al. [139] rapidly recognized three different strains of *E. coli*. The conjugated polymers bearing negative charge in the sensor systems were eventually replaced by the pathogenic cells which differentially restored the polymer fluorescence.

Nanotechnology offers novel prospects for redefining the constraints of human discernment. In the course of evolution, the olfactory system of human beings has got the unique ability to detect volatile organic compounds present at tremendously low concentrations in different complex environments [140]. The great sensitivity and flexibility of human beings to differentiate more than a trillion olfactory stimuli marks olfaction as an encouraging dais for different biotechnological applications [141, 142]. Various effective sensors that primarily function based on olfaction have been proposed for unveiling bacteria. The system of such nanosensors is mainly encompassed of three different constituents: 1) surface-functionalized nanoparticles, 2) pro-smell fragments, and 3) enzymes that slice the pro-fragrances for generating the olfactory output. The fine-tuning of these three components offer a delicate sensory system, which allows the rapid detection of bacteria at levels as low as 10^2^ CFU/ML [143]. The introduction of magnetic nanoparticles also enables the separation, purification, and recognition of pathogens under complex environments. The nanomaterial-grounded, ‘enzyme nose’ nanosensor is also a convenient investigative method meant for detecting toxicologically significant targets present in natural samples. Sun et al. [134]designed a unique enzyme nanosensor, which was grounded on the non-covalent centers, for detecting pathogens. The employment of magnetic nanoparticles–urease sensors permitted the profound recognition of bacteria with a precision of 90.7% at the concentration of 10^2^ CFU/LL in a very small time of 30 min. Similarly, various other different types of optical, electrochemical, and immunosensors have also been developed for detecting diverse plant pathogenic microorganisms (Table 3). For instance, the optic particle plasmon resonance immunosensor synthesized using gold nanorods effectively detected *Cymbidium mosaic virus* (CymMV) or *Odontoglossum ringspot virus* at the concentrations of 48 and 42 pg/mL (Lin et al. 2014) whereas the Fe_3_O_4_/SiO_2_ based immunosensor revealed the presence of *Tomato ringspot virus*, *Bean pod mottle virus* and *Arabis mosaic virus* at the concentrations of 10^−4^ mg/mL [144]. Therefore, directing the performance of approachable nanomaterials at the molecular scale can be exploited to revise the annotations of humans regarding their environments in a fashion that seems otherwise unmanageable.

**Table 3:** Review of literature illustrating the nanosensors for detecting other entities

Nanosensor type	nanomaterial used	Entity detected/purpose	Limit of detection	Method of nanosensor formulation	Sensing mechanism	Observation	References
Optic particle plasmon resonance (FOPPR) immunosensor	Gold nanorods	*Cymbidium mosaic virus* (CymMV) or *Odontoglossum ringspot virus* (ORSV)	48 and 42 pg/mL for CymMV and ORSV respectively	Gold nanorods were synthesized using the seed-mediated growth method followed by their immobilization on the fiber core surface. Afterward, the AuNR surface was functionalized by antibodies through sinking the fiber in a solution of CymMV or ORSV antibody	Detection strategy is based on the localized evanescent field absorption by the AuNRs upon biomolecular binding which results in decreased transmission intensity measured at the distal end of the fiber	This nanosensor solves the problem of color interference encountered by using AuNSs, provides faster analysis, better reproducibility, and lower detection limit	[184]
Nanoparticles based immunosensor	Fe_3_O_4_/SiO_2_	Tomato ringspot virus (ToRSV), bean pod mottle virus (BPMV) and arabis mosaic virus (ArMV)	10^−4^ mg/mL	Metal nanoparticles were surface modified to form amino-functionalized Fe_3_O_4_/SiO_2_ MNPs (NH_2_-Fe_3_O_4_/SiO_2_ MNPs) followed by the covalent immobilization of antibody	There is a good linear relationship between the enhanced fluorescence and the concentrations of viruses	The target viruses can be detected by a nongrowth step	[144]
Electrochemical sensor	Multiwalled carbon nanotube	*Ganoderma boninense*	0.0414 mg/L	Gold (III) chloride trihydrate and sodium citrate dehydrate used for the synthesis of gold nanoparticles of different sizes and layer by layer assembly was used to modify the electrode	Electrocatalytic activities of a modified electrode towards oxidation of healthy and *G. boninense*-infected oil palm leaves	A sensitivity and reproducible method due to the unique characteristics of nanoparticles	[185]
Electrochemical nanosensor	TiO_2_ or SnO_2_ nanoparticles on screen-printed carbon (SP) electrodes	*Phytophthora cactorum*	35–62 nM	SnO_2_ and TiO_2_ were used as electrochemical detection elements for amperometric sensing and Screen-printed carbon electrodes were modified with nanoparticles of SnO_2_ or TiO_2_ before their use in electrochemical detection	Detection of symbolic volatile compound p-ethyl guaiacol produced during infection	Electroanalytical data obtained using cyclic voltammetry and differential pulse voltammetry exhibited that both SnO_2_ and TiO_2_ displayed high sensitivity	[186]
Electrochemical Biosensor	Colloidal Gold Nanoparticles	*Pseudomonas syringae*	214 pM	AuNPs were synthesized by the citrate reduction of HAuCl_4_. The AuNP-DNA probe was prepared by adding tris(2-carboxyethyl)phosphine to DNA mixture of *Pseudomonas syringae*	Assessment of electrochemical changes with differential pulse voltammetry	This method can readily identify P. syringae infected plant samples even before the appearance of disease symptoms	[187]
Optical nanosensors	Selective single-walled carbon nanotube	Wound signaling	-	Cy3-labeled G-SWNT was prepared by mixing 1 mg of Cy3-ss(GT)15 and 0.25 mg of HiPCO SWNT, followed by the purification	Selective single-walled carbon nanotubes are excellent fluorescent probes that have the capability for real-time monitoring of H_2_O_2_ produced due to mechanical injury in plants	This nanosensor probe is independent of species and capable of real-time, spatial and temporal biochemical measurements in plants	[188]
Oxygen nanosensors	Carbon-filled quartz micropipettes having platinum-coated tips	Oxygen concentration	-	The inert carbon surface of the electrode was functionalized for the detection of redox-active species. A nanocavity was created in a carbon electrode. The fabrication of platinum nanosensors was performed in two stages: etching in alkaline solution followed by the platinization	The platinized nanoelectrode displays enhanced catalytic activity for oxygen reduction and the current of the sensor electrode was recalculated to oxygen concentration	Such novel platinum nanoelectrodes are beneficial for understanding cell oxygen metabolism	[189]
Fluorescent nanosensor	Carbon dots (CDs)	Fe^3+^	6.4 nM	CDs were synthesized from Pseudo-stem of banana plant (as carbon source) by using hydrothermal method	Drastic decrease in the fluorescence intensity of CDs upon increase in the Fe^3+^ concentration	CDs are highly selective to Fe3 + ions even in the presence of other ions	[190]
SERS-barcoded nanosensor	Au	Bacillus thuringiensis (Bt) gene transformed rice expressing insecticidal proteins	0.1 pg/mL	Encapsulation of gold nanoparticles with silica and conjugation of oligonucleotide strands for targeting DNA strands	DNA hybridization	The nanosensor provides precise detection of transgenic rice varieties	[163]
SPR nanosensor	Au	Aflatoxin B1	1.04 pg mL^−1^	Aflatoxin and N-methacryloyl-L-phenylalanine were pre-complexed as a template molecule and functional monomer. Molecularly imprinted polymers with gold nanoparticles were coated onto surface plasmon resonance (SPR) gold chip surface	The reflectivity index in the gold electrode surface changed with the aflatoxin concentration	SPR nanosensors were have commendable selectivity and reusability	[162]

## Nanosensors for Detection of Other Entities

Amino acids are very crucial molecules required by the living systems as they play a pivotal role of building blocks in the process of protein synthesis [145], vital character for maintenance of redox environments in the cell and extenuating destruction from the toxin and free radicals [146]. The investigative methods for detecting amino acids have been reported, especially by chromatography, chemiluminescence, and electrochemistry [147]. However, the application of existing technologies is greatly restricted by the great expenses and time-consuming steps. Currently, nanomolecular sensors have been established for detecting such molecules owing to their chemical steadiness, bio-compatibility, and easy surface alteration [148, 149]. The employment of gold nanoparticles for biosensing solicitations has been reported in different biological environments. The amine side chain and sulfhydryl (thiol) group of amino acids may perhaps covalently bind with the gold nanoparticles, thereby inducing an accretion of these nanostructures which further results in a color alteration from red to blue on the aggregation of amino thiol molecules [150, 151]. Chaicham et al. [147] developed an optical nanosensor grounded on gold nanoparticles that could detect Cys and Lys at concentrations of 5.88 μM and 16.14 μM, respectively, along with an adequate percentage retrieval of 101–106 in actual samples.

Similarly, other metal ions that are required by living organisms for performing various metabolic functions can be detected by employing different nanosensors. A dual-emission fluorescent probe was developed by Lu et al. [152] for detecting Cu^2+^ ions by condensing hydrophobic carbon dots in micelles molded by the auto-assemblage of different amphiphilic polymers. A vigorous, self-accelerating, and magnetic electrochemiluminescence nanosensor which was established on the multi-functionalized CoFe_2_O_4_ MNPs was established for the foremost and later employed for the extremely sensitive as well as discriminating recognition of the target Cu^2+^ through click reaction in a quasi-homogeneous system [82]. Gold nanorods are also exploited for sensing Fe (III) ions. Thatai et al. [17] devised highly sensitive gold nanorods using cetyltrimethylammonium bromide as illustrative material for detecting ferric ions along with a surprising sensing level equivalent to 100 ppb. Zinc is another important element, and it occurs in a divalent cationic form as Zn^2+^ ions. Zn^2+^ ion has the capability of sustaining important activities counting synthesis of DNA and protein, RNA transcription, cell apoptosis, and metalloenzyme regulation [153, 154]. Usually, fluorescent probes are exploited for detecting the Zn^2+^ ions in biological systems. The pyridoxal-5′-phosphate (PLP) conjugated lysozyme cocooned gold nanoclusters (Lyso-AuNCs) can also be exploited for the selective and turn-on detection of divalent Zn^2+^ ions in the liquid environment. The yellow fluorescence of PLP Lyso-AuNCs displays noteworthy augmentation at 475 nm in the occurrence of Zn^2+^ generating bluish-green fluorescence which is accredited to the complexation-induced accretion of nanoclusters. The developed nanoprobe can detect Zn^2+^ ions in nanomolar concentrations (39.2 nM) [154]. The dual-emission carbon dots (DCDs) synthesized by Wang et al. [155] can also be exploited for revealing Zn^2+^ ions as well as iron ions (Fe^3+^) in different pH environments. The ferric ions could also be detected in an acidic environment along with an amazing sensation level equaling 0.8 µmol/L while Zn^2+^ ions could be detected in an alkaline environment along with a detection limit of 1.2 µmol/L.

These days groundwater is used for irrigation and it is also the solitary seedbed of potable water in numerous regions, exclusively in the isolated agronomic sections. The capricious expulsion of numerous contaminants into the environment has expressively deteriorated the eminence of groundwater, thus has significantly threatened environmental safety [156, 157]. Although there are numerous micropollutants, however, the rushing of fluoride in groundwater has stretched out accumulative civic consideration as a result of the grave fluorosis, severe abdominal and renal complications persuaded by the elevated intake of fluoride ion [158]. So, there is a quest to diagnose and unveil hardness as well as the presence of fluoride ions in the ground-water which has expected substantial considerations owing to their significant parts in the different ecological, biological, and chemical processes [157]. Although fluorescent probes which are considered as traditional methods, can be exploited for detecting F^−^, however, the employment of quantum dots, an inorganic nanomaterial, can grab extensive considerations on account of their distinctive optical possessions comprising size-oriented fluorescence, tapered and coherent emission peak with a wide exciting wavelength, and outstanding photo solidity [159, 160]. The creation of a fluorescence resonance energy transmission channel from the carbon dots and the gold nanoparticles appears to be a competent solution for detecting numerous analytes. Therefore, constructing a novel nanosensor via gold nanoparticles and carbon dots for detecting F^−^ seems to be a proficient strategy. The hybrid nanosensor assorted with calcium ions has been reported to spot fluoride ions along with a subordinate recognition level parallel to 0.339 ppm [103]. Lu et al. [161] also developed another novel strategy for detecting fluoride, which was grounded on dual ligands coated with perovskite quantum dots, and the recognition level was found to be 3.2 μM.

The agricultural systems also necessitate the diagnosis of various other entities for the smooth functioning and enhanced productivity of the agroecosystems. The detection of other miscellaneous entities has also been facilitated by the employment of nanosensors (Table 3), for instance, the detection of transgenic plants, the presence of aflatoxins, and even the occurrence of wounds in plants. The SPR nanosensor developed using gold nanoparticles detected the Aflatoxin B1 at the concentration of 1.04 pg mL^−1^ [162] whereas the SERS-barcoded nanosensor fabricated using the encapsulation of gold nanoparticles with silica followed by the conjugation of oligonucleotide strands effectively detected the presence of *Bacillus thuringiensis* (Bt) gene-encoded insecticidal proteins in rice plants at 0.1 pg/mL, thereby, clearly advocating the transgenic nature of rice plants [163].

## Nanosensors for Detection of Nanoparticles

Nanomaterials can also occur naturally, such as humic acids and clay minerals; extensive human activities can also lead to the incidental synthesis of various nanomaterials in the environment, for instance, diesel oil emanations or by the discharge of welding fumes; or they can also be explicitly concocted to unveil matchless electrical, optical, chemical or physical features [164]. These characteristics are exploited in plenty of consumable merchandise, for instance, medicines, food, cosmetics and suntan lotions, paints, and electronics, as well as processes that directly discharge nanomaterials into the surroundings, such as remediating contaminated environs [165, 166]. Furthermore, the rapid employment of metal nanoparticles in various systems has raised many concerns due to the potential environmental risks posed by them as they are unavoidably lost in the environment throughout the processes meant for their fabrication, conveyance, usage, and dumping [167]. Carbon-based nanomaterials are quite established against degradation and as a result, amass in the surroundings [168]. Nanoparticles, attributable to their greater surface area, find it much easier to bind and adsorb on the cellular surfaces. They harm the cell in several ways, such as, by hindering the protein transport pathway on the membrane, by destroying the permeability of the cell membrane, or by further inhibiting core components of the cell [169]. Currently, an overwhelming figure of the engineered nanoparticles engaged for different ecological and industrial solicitations or molded as by-products of different human deeds are ultimately discharged into soil systems. The usual nanoparticles employed comprise the metal engineered nanoparticles (elemental Fe, Au, Ag, etc.), metal oxides (SiO_2_, ZnO, FeO_2_, TiO_2_, CuO, Al_2_O_3_, etc.), composite compounds (Co–Zn–Fe oxide), fullerenes (grouping Buckminster fullerenes, nanocones, carbon nanotubes, etc.), quantum dots frequently encrusted with a polymer and other organic polymers (Dinesh et al. 2012). Different plant growth-promoting rhizobacteria (PGPR) like *Bacillus subtilis*, *Pseudomonas aeruginosa*, *P. fluorescens, *and *P. putida*, and different bacteria involved in soil nitrogen transformations are inhibited to varying degrees on exposure to nanoparticles in aqueous suspensions or pure culture conditions [170]. The nanoparticles grounded on metals copper and iron are alleged to interact with the peroxides existing in the environs thereby engender free radicals that are notorious for their high toxicity to microbes [171]. Therefore, there is a strong need to monitor the different nanoparticles which find an ultimate sink in the soils especially of agroecosystems.

Various techniques can be reconnoitered for sensing nanoparticles, one among them is the usage of microcavity sensors, which, in the form of whispering gallery resonators have acknowledged extensive consideration. Here, the particle binding on the exterior of the microcavity disturbs the optical possessions thereby instigating a resonant wavelength swing with magnitude reliant upon the polarizability of the particle. The measure of the change facilitates surveillance of the binding actions in real-time and is also used to evaluate the particle size [172]. Optical sensing empowered with the extreme sensitivity of single nanoscale entities is sturdily anticipated for solicitations in numerous arenas, for instance, in environmental checking, other than in homeland security. Split-mode microcavity Raman lasers are also highly sensitive optical sensors that can perceive the occurrence of even a single nanoparticle. The presence of nanoparticles is revealed by observing the distinct alterations in the beat frequency of the Raman lasers and the sensing level has been reported to be 20 nm radius of the nanoparticles [138].

## Nanotechnology Implementation in an Agroecosystem: Proof-of-Concept to Commercialization

There are hundreds of research articles and studies that are being published every year on nanosensor's application in agriculture. However, very few nanosensors have yet been commercialized for the detection of heavy metals, pesticides, plant-pathogen, and other substances in an agroecosystem. Because these academic outputs are not properly converted/conveyed to commercial or other regulatory platforms. Certain scientific and non-scientific factors hinder these nanosensors from proof-of-concept to fully commercialized products. These factors are scale-up and real-use (technical), validation and compliances (regulatory), management priorities and decisions (political), standardization (legal), cost, demand and IPR protection (economic), safety and security (environmental health and safety) along with several ethical issues. It is necessary to support enthusiastic researchers and institutions for research and development to develop such nanosensors for agroecosystem, product validation, intellectual protection, and their social understanding and implementation. If we consider these factors strategically, it will help in nanosensor product betterment and implementation to agroecosystem. The US-based startup Razzberry developed portable chemical nanosensors to trace real-time chemical changes in water, soil, and the environment. Similarly, Italian startup Nasys invented a metal oxides-based nanosensor to detect air pollution. There are some other startups nGageIT and Tracense, implementing nanosensor technologies to detect biological and Hazardous contaminants in agriculture.

## Perspectives and Conclusions

Since times immemorial, agriculture is the main source of food, income as well as employment for mankind around the globe. In the present era, due to upsurge of rapid urbanization and climate inconsistency, precision farming has been flocking significant attention worldwide. In agricultural system, this type of farming has the ability to maximize the crop’s productivity and improve soil quality along with the minimization of the agrochemicals input (such as fertilizers, herbicides, pesticides, etc.). Precision farming is possible through focused monitoring of environmental variables along with the application of the directed action. This type of farming system also employs computers, global satellite positioning systems, sensors, and remote sensing strategies. As a result, the monitoring of extremely confined environmental situations becomes easy. This monitoring even assists in defining the growth of crop plants by accurately ascertaining the nature and site of hitches. Eventually, it also employs smart sensors for providing exact data that grant enriched productivity by serving farmers to make recovery choices in a detailed manner. Among all the sensors, smart nanosensors are very sensitive and judiciously employed devices that have started proving to be an essential tool for advocating agricultural sustainability, in future.

It has been noticed that the use of nanosensors and or biosensors can accelerate agricultural productivity. These real-time sensors can physically monitor temperature, soil health, soil moisture content and even senses the soil microbiological/microenvironment and nutrient status of soils. Interestingly, these sensors have also been able to detect residual pesticides, heavy metals, monitor plant pathogens and quantify fertilizers and toxins. These nanosensors facilitate speedy, quick, reliable, and prior information that even aid in predicting as well as mitigating the crop losses in the agroecosystems. In addition, the use of nanotechnology-based biosensors also assists in accomplishing the concept of sustainable agriculture. It has been observed that the projection of nanosensors and or biosensors as plant diagnostic tools requires improvements regarding their sensitivity and specificity. Additionally, there is a need for quick, reliable, cheap, multiplexed screening to detect a wide range of plant-based bioproducts. Moreover, the development of broad-spectrum nanosensors that can detect multiple entities will also boost in mobilizing technology. It has been suggested that the biosensor efficiency can be improved further by developing super “novel nanomaterials” that will be available in near future. Perhaps in the coming years, the convergence among nanotechnology, agriculture sciences, rhizosphere engineering, and overall plant engineering will lead to the path towards accomplishment of all Sustainable Development Goals 2030 without incurring any fitness cost on mankind safety, economy, natural resources, and environment.

## Data Availability

Not applicable.

## Abbreviations

AAS: Atomic absorption spectrometry
AES: Atomic emission spectrometry
Ag: Silver
Al_2_O_3_: Aluminum oxide
Au: Gold
CdSe: Cadmium selenide
CL: Colorimetric
CoFe_2_O_4_: Cobalt iron oxide
CTBr: Cationic cetyltrimethylammonium bromide
CuO: Cupric oxide
DCDs: Sual-emission carbon dots
FeO_2_: Iron dioxide
FL: Fluorescence
ICP-MS: Inductively coupled plasma mass spectrometry
ICTS: Immunochromatographic strip
NiO: Nickel oxide
Pd: Palladium
PGPR: Plant growth-promoting rhizobacteria
SERS: Surface-enhanced Raman scattering
SiO_2_: Silicon dioxide
SPR: Surface plasmon resonance
TiO_2_: Titanium dioxide
XRF: X-ray fluorescence spectrometry
ZnO: Zinc oxide
ZrO_2_: Zirconium dioxide
